# Characteristics, Regional Evaluation, and D-Antigen in Transfusions by Emergency Medical Services

**DOI:** 10.1001/jamanetworkopen.2025.24368

**Published:** 2025-07-31

**Authors:** Carlo L. Rosen, Sarah Alice Thomas, Michael P. McCartin, Kerry L. O’Brien, Ira J. Blumen, Kathie Dunn, Thomas Judge, Michael M. Choate, Stephan E. Russ, Jeff Goodloe, Anna Bailey, Jeremy F. Norman, Denise Bäckström, Jason Cohen, Matt Donohue, Christie L. Fritz, David W. Schoenfeld, Stephen H. Thomas

**Affiliations:** 1 Department of Emergency Medicine, Beth Israel Deaconess Medical Center, Harvard Medical School, Boston, Massachusetts; 2 Department of Molecular Microbiology and Immunology, Johns Hopkins Bloomberg School of Public Health, Baltimore, Maryland; 3 Section of Emergency Medicine and University of Chicago Aeromedical Network, University of Chicago, Chicago, Illinois; 4 Department of Pathology, Beth Israel Deaconess Medical Center, Harvard Medical School, Boston, Massachusetts; 5 Global Medical Response, Lewisville, Texas; 6 LifeFlight of Maine, Bangor; 7 Vanderbilt LifeFlight, Department of Emergency Medicine, Vanderbilt University, Nashville, Tennessee; 8 Department of Emergency Medicine, University of Oklahoma School of Community Medicine, Tulsa; 9 Office of the Medical Director, EMS System for Metropolitan Oklahoma City and Tulsa, Oklahoma City and Tulsa, Oklahoma; 10 Air Methods LLC, Greenwood Village, Colorado; 11 Department of Biomedical and Clinical Sciences, Linköping University, Linköping, Sweden; 12 Department of Leadership and Command and Control, Swedish Defense University, Karlstad, Sweden; 13 Boston MedFlight, Bedford, Massachusetts; 14 Division of Trauma, Burns, and Surgical Critical Care, Department of Surgery, Brigham and Women’s Hospital, Boston, Massachusetts; 15 Blizard Institute for Neuroscience, Surgery, and Trauma, Barts and The London School of Medicine, London, UK

## Abstract

**Importance:**

Prehospital transfusion (PHT) of blood products by emergency medical services (EMS) has become common in civilian settings. However, variability exists in practices across the US. There are few large-scale data describing US civilian PHT with regard to blood products administered, potential exposure of females of childbearing potential to D-positive blood, or the proportion of PHT cases occurring in EMS transports of short duration.

**Objective:**

To evaluate nationwide PHT trends, regional differences, and potential risks, particularly for D-positive blood administration in females of childbearing potential.

**Design, Setting, and Participants:**

This cohort study, Characteristics, Regional Evaluation, and D-Antigen in Transfusions by EMS (CREDIT-EMS), assessed data collected from January 1, 2020, to October 31, 2024 (before hypothesis generation), in trauma and nontrauma patients of all ages treated in 48 states in the US.

**Exposure:**

Prehospital-initiated transfusion of blood products.

**Main Outcomes and Measures:**

Age, sex, blood product characteristics (including blood type), and transport time. Comparative analyses were conducted using Pearson χ^2^, Wilcoxon rank sum, and nonparametric trend tests. Proportions were reported with binomial exact 95% CIs.

**Results:**

The study analyzed 10 444 patients (median [IQR] age, 45 [29-63] years; 7302 of 10 439 [70.0%] male) who received 18 177 units of blood products. The proportion of transfused units that were whole blood (WB) increased from 10.0% in 2020 to 30.8% in 2024 (*P* < .001). Regional variations in PHT were identified, with the Northeast having the highest proportion of WB use (33.2% of PHT). A total of 1589 (15.2%; 95% CI, 14.5%-15.9%) of the study patients were females of childbearing potential (aged 12-50 years), with an increasing number receiving D-positive blood products over time (73 of 169 [43.2%] in 2020 to 372 of 497 [74.9%] in 2024) (*P* < .001). Prehospital times were 20 minutes or less in only 255 of 10 343 cases (2.5%; 95% CI, 2.2%-2.8%).

**Conclusions and Relevance:**

This cohort study of civilian PHT practices in the US found geographic and temporal variability. There was increasing adoption of WB and significant implications for females of childbearing potential who are increasingly likely to receive D-positive PHT. Cases of PHT uncommonly involved short prehospital duration. These findings highlight the need for standardized protocols and further evaluation of risk-benefit considerations.

## Key Points

**Question:**

What are the patterns and regional trends of prehospital blood transfusions in civilian emergency medical services settings across the US?

**Findings:**

This cohort study of 10 444 patients identified a significant increase in whole blood use (from 10% to 30%), along with regional differences in transfusion practices. In female patients of childbearing potential there was a marked increase (from 43% to 75%) in the proportion of emergency medical services–transfused blood that was D-positive.

**Meaning:**

These findings highlight current US practice and trends while underscoring the importance of standardizing transfusion protocols.

## Teaser

This cohort study of patients receiving blood products examines geographic variations in prehospital transfusions by emergency medical services (EMS) in different areas of the US.

## Introduction

Prehospital transfusion (PHT) is moving from its roots in military medicine^1^ to increasingly widespread application in the civilian setting.^2,3,4^ As defined in recent reviews,^5,6^ PHT denotes administration of whole blood (WB) or any of its components, including red blood cells (RBCs), plasma, cryoprecipitate, and platelets, by emergency medical services (EMS). Results from a 2024 meta-analysis^6^ comprising data from the only 3 randomized clinical trials (RCTs)^7,8,9^ of nonmilitary PHTs suggested, but did not statistically confirm, a 1-month survival benefit.^6^ A second 2024 meta-analysis,^10^ not restricted to RCTs, did not identify a 1-month survival benefit to RBCs transfused with or without plasma.

Plasma was used in the Prehospital Plasma During Air Medical Transport in Trauma Patients at Risk for Hemorrhagic Shock (PAMPer)^9^ and Control of Major Bleeding After Trauma Trial (COMBAT).^8^ In the Resuscitation with Blood Products in Patients With Trauma-Related Hemorrhagic Shock Receiving Prehospital Care (RePHILL) trial,^7^ participants received up to 2 units each of lyophilized plasma and RBCs. The Type O Whole Blood and Assessment of Age During Prehospital Resuscitation (TOWAR)^11^ and the Trauma Resuscitation With Low-Titer Group O Whole Blood or Products (TROOP)^12^ evaluate WB vs blood components or crystalloid, whereas Prehospital Transfusion Strategy in Bleeding Patients (PRIEST)^13^ evaluates RBC and/or plasma. Another trial, the Study of Whole Blood in Frontline Trauma (SWIFT),^14^ evaluates WB vs component therapy.

The array of PHT approaches in the completed and ongoing RCTs prompts the study question we address: “What is currently occurring in everyday EMS transfusion practice?” It has been suggested that the treatment arms evaluated by available RCT data do not match typical US PHT practice.^15,16^ However, despite substantial discussion and debate regarding civilian PHT,^5,16,17,18,19,20^ few, if any, data precisely describe the evolution and current state of routinely practiced EMS transfusion in the US.

One of the key areas for inquiry in the realm of PHT is the potentially increasing administration of D-positive WB or RBC to females of childbearing potential who may be D-negative.^21^ If a D-negative FCP receives D-positive RBCs, risks include alloimmunization and hemolytic disease of the fetus and newborn (HDFN) affecting future pregnancies. The risk appears to be equal in the formulations of RBCs and WB used in civilian PHT programs and with transfusion of one unit vs multiple units.^22,23^ Risk is modified by patient age, with higher risk with younger age at time of alloimmunization.^24^ HDFN-related patient discussions, diagnostic evaluation, preventive treatment, and related care are associated with patient risk and resource use.^19,25^ Alloimmunization risk should neither block life-saving transfusion nor be dismissed when considering PHT costs and benefits. Alloimmunization risks should also be considered when determining blood products used for the PHT population.

The current study, Characteristics, Regional Evaluation, and D-Antigen in Transfusions by EMS (CREDIT-EMS), was undertaken to provide improved understanding of civilian PHT in the US, with basic description of nationwide practices and demographics. We also set out to ascertain whether there were geographic PHT variations between different areas of the US because previous work^26^ suggests geographic variation in patient perceptions about PHT. Our goal was to facilitate discussion and modeling of PHT by reporting on a large, nationwide dataset of EMS-initiated transfusions.

## Methods

### Design, Setting, and Data Sources

This study was an observational (noninterventional) retrospective cohort study of prehospital data. This investigation was determined to meet the criteria for exempt status under category 4 and was granted a HIPAA waiver of authorization by the Beth Israel Deaconess Medical Center Committee on Clinical Investigations. The study followed the Strengthening the Reporting of Observational Studies in Epidemiology (STROBE) reporting guideline.^27^

CREDIT-EMS used 6 data sources that, when combined, represented more than 500 EMS services providing PHT nationwide. Geographic classification was based on the 4 US Census Bureau regions (eFigure 1 in Supplement 1). EMS program types, staffing patterns, and transfusion protocols varied widely across the study sources. Although some national-level corporate databases were used for study information, the EMS each had their own medical direction and protocols. eAppendix 1 in Supplement 1 provides further information on participating EMS.

Data came from advanced life support and critical care transport services. Two data sources were nationwide commercial entities, which together operate nearly 500 primarily air EMS bases. The other 4 data sources included 3 nonprofit primarily air EMS programs (based in Maine, Massachusetts, and Tennessee) and an Oklahoma public service ground EMS program.

### Patients

CREDIT-EMS defined civilian PHT as administration of WB or any component in the prehospital setting of air or ground ambulance response to scenes (ie, not interfacility transports). Civilian PHT as reported here includes only transfusions initiated by the participating EMS. Plasma was defined to include fresh frozen plasma, liquid plasma, and cryoprecipitate.

Patients of any age were eligible if their cases were scene transports for any diagnosis from January 1, 2020, through October 31, 2024. Data were collected before hypothesis generation. Patients were classified as females of childbearing potential if they were females aged 12 to 50 years (as collected by the transport services and recorded in their electronic transport records). Eligibility criteria included patients transported by any vehicle and with any crew configuration as long as transfusion of a blood component was initiated by EMS in the field. Patients were excluded if transfusion was planned but not initiated. Data on patient race and ethnicity were not collected because these data elements were not consistently available across study sites.

### Statistical Analysis

The main unit of analysis was the patient (transfusion recipient). Data were analyzed using Stata software, version 18.5MP (StataCorp), which was used for all calculations and plotting. Significance was defined at a 2-sided *P* < .05, and 95% CIs are reported.

Descriptive statistics for categorical data reported proportions with binomial exact 95% CIs. Univariable analyses used Pearson χ^2^ testing, with use of the Fisher exact test if any cell value was 5 or less. Effect size estimation was reported as risk ratios (RRs) with 95% CIs.

For continuous data, which were found nonnormal by quantile-normal plotting, descriptive reporting assessed the median (IQR). For key findings, the Stata rank-based conservative approach calculated 95% CIs for medians.

Continuous variables were first evaluated for significant differences among groups (eg, US regions) using Kruskal-Wallis testing. For comparisons involving 3 or more groups, post hoc pairwise comparisons were executed using the Dunn test. Effect estimates were calculated as median intergroup differences using the Stata Hodges-Lehmann estimation of median between-group differences. Where trends across ordered groups (eg, years) were evaluated, we used Cochran-Armitage or Cuzick trend testing.

## Results

### Characteristics of Transfusion Recipients

The study accrued 10 444 patients (median [IQR] age, 45 [29-63] years; 7302 of 10 439 [70.0%] male and 3137 of 10 439 [30.1%] female) with civilian PHT of 1 or more units of blood products transfused. Recipients of PHT were identified from 48 US states; all states were represented in the CREDIT-EMS population except for Delaware, Rhode Island, and the District of Columbia. A state-based overview of study representation is shown in eFigure 2 in Supplement 1.

A total of 8419 patients (80.6%) had a trauma diagnosis, and 10 177 (97.4%) were transported by air. Median (IQR) duration of prehospital time for the 10 343 cases (99.0%) with available data was 48 (36-64) minutes. Prehospital times were 20 minutes or less in 255 of 10 343 cases (2.5.%; 95% CI, 2.2%-2.8%). Additional details on prehospital times are provided in the eResults and eTable 1 in Supplement 1.

The outcome of survival to hospital discharge was recorded by 3 sources, all of which reported data on 100% of their cases (N = 1830). The survival rates were 97.2% (95% CI, 96.3%-97.9%), 71.6% (95% CI, 60.5%-81.1%), and 68.8% (95% CI, 41.3%-89.0%) in the 3 sources. Other characteristics of the study population are given in Table 1. For the site contributing data in 2023 and 2024, there was no PHT protocol until 2023.

**Table 1.** Characteristics of Prehospital Transfusion Recipients

Characteristic	Patients, No./total No. (%)^a^
Data source^b^ (calendar years of data contributed)	
A (2024)	1733/10 444 (16.6)
B (2023 and 2024)	81/10 444 (0.8)
C (2020-2024)	8319/10 444 (79.7)
D (2020-2023)	89/10 444 (0.9)
E (2023 in the US)	206/10 444 (2.0)
F (2020-2024)	16/10 444 (0.2)
Year	
2020	1189/10 444 (11.4)
2021	1569/10 444 (15.0)
2022	1750/10 444 (16.8)
2023	2652/10 444 (25.4)
2024	3284/10 444 (31.4)
Geographic location	
Northeast	334/10 443 (3.2)
Midwest	1472/10 443 (14.1)
South	7781/10 443 (74.5)
West	856/10 443 (8.2)
Demographics	
Age, median (IQR) [range], y	45 (29-63) [1-104]
Sex	
Male	7302/10 439 (70.0)
Female (aged 12-50 y)	1589/10 438 (15.2)
Female (not aged 12-50 y)	1547/10 438
Prehospital emergency medical services vehicle	
Ground ambulance	267/10 444 (2.6)
Fixed wing (airplane)	144/10 444 (1.4)
Rotor wing (helicopter)	10 033/10 444 (95.1)

^a^ Unless otherwise indicated.

^b^ Each letter (A–F) represents a distinct deidentified emergency medical system or helicopter emergency services system contributing to the CREDIT-EMS dataset. System-level identifiers were anonymized for analysis.

Geographic characteristics were assessed based on US Census Bureau region (of receiving hospital). Of 10 444 cases, there was only 1 geographic region involved (with EMS agency response location and receiving hospital location) in 10 045 cases (96.2%). Of 10 444 US cases, age was missing in 2 cases (one of whom was female and thus potentially an FCP), and sex was missing in 5 cases (all in the 12- to 50-year age range and thus potentially females of childbearing potential). There were 10 438 cases with known FCP status.

### Characteristics of Transfusions

The 10 444 patients had a total of 17 927 units of various types of blood product transfusions (eFigure 3 in Supplement 1) initiated in the prehospital setting. Of the 10 444 recipients of PHT (Table 2; eFigures 4-6 in Supplement 1), 9185 (88.0%) were administered WB and/or RBCs. Two or more units of blood product were initiated in 5000 of 10 444 patients (47.9%; 95% CI, 46.9%-48.8%). At least 1 unit was fully transfused before hospital arrival in 8366 of 10 444 cases (80.1%; 95% CI, 79.3%-80.9%); 2 or more units were completed in 3911 of 10 444 cases (37.5%; 95% CI, 36.5%-38.4%).

**Table 2.** Characteristics of Transfusions

Characteristic	Patients, No./total No. (%)
Patients receiving blood product types (any number of units)	
Whole blood	2122/10 444 (20.3)
Red blood cell	7177/10 444 (68.7)
Plasma	4750/10 444 (45.5)
Platelet	44/10 444 (0.4)
No. of transfused units initiated in prehospital setting	
1	5444/10 444 (52.1)
2	3438/10 444 (32.9)
3	721/10 444 (6.9)
4	792/10 444 (7.6)
5	30/10 444 (0.3)
>5	19/10 444 (0.2)
No. of transfused units in patients with transfusions completed before hospital arrival	
0	2078/10 444 (19.9)
1	4455/10 444 (42.7)
2	2660/10 444 (25.5)
3	588/10 444 (5.6)
4	631/10 444 (6.0)
5	21/10 444 (0.2)
>5	11/10 444 (0.1)
No. of transfused units in females aged 12-50 y receiving D-positive whole blood or red blood cells	
0	632/1589 (39.8)
1	739/1589 (46.5)
2	201/1589 (12.7)
3	13/1589 (0.8)
4	4/1589 (0.3)

Of the 10 438 patients with known FCP status, 1589 (15.2%; 95% CI, 14.5%-15.9%) met predefined criteria as females aged 12 to 50 years. In these 1589 females of childbearing potential, 957 (60.0%; 95% CI, 57.8%-62.6%) received at least 1 unit of D-positive WB or RBCs (eFigure 7 in Supplement 1), and females of childbearing potential received 2 or more D-positive WB or RBC units in 218 cases (13.7%; 95% CI, 12.1%-15.5%).

D-positive exposure was more common in patients receiving WB compared .with those receiving only RBCs. Of the 1589 females of childbearing potential, either WB or RBCs were administered in 1393 (87.7%). In these 1393 patients, 957 (68.7%) of whom received at least 1 unit of D-positive WB or RBC, the relative proportion of receipt of D-positive blood product was significantly higher in those receiving WB compared with the reference group of those not receiving WB (RR, 1.53; 95% CI, 1.44-1.62; *P* < .001).

### Analysis for Regional Differences

Table 3 presents the results of comparisons among regions within the US. Transfusion practices varied across regions. The Figure illustrates state-based WB use. As compared to the baseline West region, WB was more likely (*P* < .004 for all pairwise comparisons) in the Northeast (RR, 2.01; 95% CI, 1.67-2.44), South (1.21; 95% CI, 1.07-1.36), and West (1.55; 95% CI, 1.32-1.82). The Northeast had the highest proportion of WB use at 33.2% (95% CI, 28.2%-38.6%; *P* < .001) compared with the Midwest at 16.5% (95% CI, 14.6%-18.5%; *P* < .001). WB use was also higher in the Northeast (RR, 1.67; 95% CI, 1.43-1.96; *P* < .001) and in the West (RR, 1.29; 95% CI, 1.14-1.45; *P* < .001) compared with the South. The Midwest’s lower WB use corresponded to higher RBC use (without WB) compared with the Northeast (RR, 1.14; 95% CI, 1.05-1.24), South (RR, 1.13; 95% CI, 1.09-1.16), and West (RR, 1.12; 95% CI 1.06-1.18) (all pairwise *P* < .001). Whole blood utilization varied significantly across regions, with the Northeast showing the highest proportion of WB use at 33.2% (95% CI, 28.2%-38.6%), compared with the Midwest at 16.5% (95% CI, 14.6%-18.5%; *P* < .001).

**Table 3.** Demographic and Transfusion Characteristics by US Region

Characteristic	Patients, No./total No. (%) [95% CI]^a^				*P* value
	Northeast	Midwest	South	West	
Demographics					
Age, median (IQR), y	56 (35-70)	50 (31-66)	44 (29-62)	49 (33-69)	<.001
All females	98/334 (29.3) [24.5-34.5]	471/1472 (32.0) [29.6-34.4]	2258/7776 (29.0) [28.0-30.1]	310/856 (36.2) [32.9-39.5]	<.001
Females aged 12-50 y	45/334 (13.5) [10.0-17.6]	219/1472 (14.9) [13.1-16.8]	1189/7776 (15.3) [14.5-16.1]	136/855 (15.9) [13.5-18.5]	.74
Transfusions					
Transfusion includes WB	111/334 (33.2) [28.2-38.6]	243/1472 (16.5) [14.6-18.5]	1548/7781 (19.9) [19.0-20.8]	219/856 (25.6) [22.7-28.6]	<.001
Transfusion includes RBCs (not as WB)	223/334 (66.8) [61.4-71.8]	1120/1472 (76.1) [73.8-78.2]	5250/7781 (67.5) [66.4-68.5]	584/856 (68.2) [65.0-71.3]	<.001
Transfusion includes WB and/or RBCs	314/334 (94.0) [90.9-96.3]	1332/1472 (90.5) [88.9-91.9]	6750/7781 (86.8) [86.0-87.5]	788/856 (92.1) [90.0-93.8]	<.001
>1 Unit initiated	133/334 (39.8) [34.5-45.3]	650/1472 (44.2) [41.6-46.7]	3912/7781 (50.3) [49.2-51.4]	305/856 (35.6) [32.4-38.9]	<.001
At least 1 unit completed	272/334 (81.4) [76.8-85.5]	1202/1472 (81.7) [79.6-83.6]	6196/7781 (79.6) [78.7-80.5]	695/856 (81.2) [78.4-83.8]	.23
>1 Unit completed	104/334 (31.1) [26.2-36.4]	545/1472 (37.0) [34.6-39.5]	3015/7781 (38.8) [37.7-39.8]	247/856 (28.9) [25.8-32.0]	<.001
Females of childbearing potential (n = 1589) receiving WB or RBCs					
At least 1 unit of D-positive WB or RBCs	29/45 (64.4) [48.7-78.1	121/219 (55.3) [48.4-62.0]	710/1189 (59.7) [56.9-62.5]	97/136 (71.3) [62.9-78.7]	.02
Multiple units of D-positive WB or RBCs	6/45 (13.3) [5.1-26.8]	30/219 (13.7) [9.4-19.0]	149/1189 (12.5) [10.7-14.5]	33/136 (24.3) [17.3-32.4]	.003

Abbreviations: RBCs, red blood cells; WB, whole blood.

^a^ Unless otherwise indicated.

**Figure.**
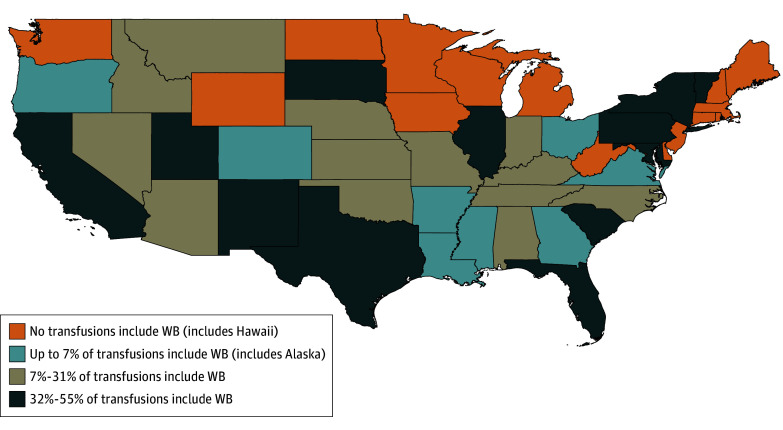
State-Based Variation in the Use of Whole Blood (WB) During Prehospital Transfusions

Table 3 indicates that WB or RBCs were used in 9185 (88.0%; 95% CI, 87.3%-88.6%) of PHT cases. Overall, plasma-only transfusions occurred in 1265 of 10 444 patients (12.1%; 95% CI, 11.5%-12.8%). Plasma-only transfusions were most common in the South (*P* < .001 for comparisons vs all other regions), occurring in 1031 of 7781 (13.3%, 95% CI, 12.5%-14.0%) PHTs (RR vs Northeast, 2.31; 95% CI, 1.49-3.59; Midwest, 1.46; 95% CI, 1.23-1.73; West, 1.85; 95% CI, 1.44-2.37). The eResults and eTable 2 in Supplement 1 contain extended findings addressing interregional differences in transfusion initiation and completion. Table 3 also gives the significant differences in regional likelihood of females of childbearing potential receiving D-positive WB or RBC. Females of childbearing potential in the West were more likely than those in the South (RR, 1.19; 95% CI, 1.06-1.34, *P* = .009) or Midwest (RR, 1.29; 95% CI, 1.10-1.51; *P* = .003) to receive at least 1 unit, and more likely than those in the South (RR, 1.94; 95% CI, 1.39-2.70; *P* < .001) or Midwest (RR, 1.77; 95% CI, 1.13-2.77; *P* = .011) to receive multiple units..

Prehospital times comparisons are detailed in the eResults and eTable 1 in Supplement 1. Even in the South, the region with the shortest median transport time, the median (IQR) prehospital time was 46 (35-61) minutes.

### Longitudinal Findings

Table 4 depicts findings for PHTs assessed by year. There were associations between study year and all assessed variables, with some associations taking the form of longitudinal trends during the 5 years of the study. Median yearly patient age ranged from 42 to 44 years for 2020 to 2022, increasing to yearly medians of 47 to 48 for the years 2023 and 2024 (*P* < .004). The eResults and eFigure 4 in Supplement 1 provide additional findings regarding yearly trends.

**Table 4.** Demographic and Transfusion Characteristics for 5 Years of US Prehospital Transfusion

Characteristic	Patients, No./total No. (%) [95% CI]^a^					*P* value
	2020	2021	2022	2023	2024	
Demographics						
Age, median (95% CI) [IQR]	44 (42-45) [28-61]	42 (40-43) [28-59]	42 (41-44) [28-60]	47 (46-48) [30-65]	48 (47-50) [31-67]	<.001
All females	320/1189 (26.9) [24.4-29.5]	466/1569 (29.7) [27.4-32.0]	469/1750 (26.8) [24.7-28.9]	815/2647 (30.8) [29.0-32.6]	1067/3284 (32.5) [30.9-34.1]	<.001
Females aged 12-50 y	169/1189 (14.2) [12.3-16.3]	285/1569 (18.2) [16.3-20.2]	256/1750 (14.6) [13.0-16.4]	382/2646 (14.4) [13.1-15.8]	497/3284 (15.1) [13.9-16.4]	.01
Transfusions						
Transfusion includes WB	122/1189 (10.3) [8.6-12.1]	196/1569 (12.5) [10.9-14.2]	326/1750 (18.6) [16.8-20.5]	500/2652 18.9) [17.4-20.4]	978/3284 (29.8) [28.2-31.4]	<.001
Transfusion includes WB and/or RBCs	1040/1189 (87.5) [85.5-89.3]	1373/1569 (87.5) [85.8-89.1]	1500/1750 (85.7) [84.0-87.3]	2360/2652 (89.0) [87.7j-90.2]	2912/3284 (88.7) [87.5-89.7]	.01
>1 Unit initiated	613/1189 (51.6) [48.7-54.4]	801/1569 (51.1) [48.5-53.6]	849/1750 (48.5) [46.1-50.9]	1278/2652 (48.2) [46.3-50.1]	1459/3284 (44.4) [42.7-46.1]	<.001
At least 1 unit completed	998/1189 (83.9) [81.7-86.0]	1306/1569 (83.2) [81.3-85.0]	1475/1750 (84.3) [82.4-86.0]	1950/2652 (73.5) [71.8-75.2]	2637/3284 (80.3) [78.9-81.6]	<.001
>1 Unit completed	497/1189 (41.8) [39.0-44.7]	676/1569 (43.1) [40.6-45.6]	705/1750 (40.3) [38.0-42.6]	872/2652 (32.9) [31.1-34.7]	1161/3284 (35.4) [33.7-37.0]	<.001
Females aged 12-50 y						
Females of childbearing potential receiving at least 1 U of D-positive WB or RBCs	73/169 (43.2) [35.6-51.0]	152/285 (53.3) [47.4-59.2]	136/256 (53.1) [46.8-59.4]	224/382 (58.6) [53.5-63.6]	372/497 (74.9) [70.8-78.6]	<.001
Females of childbearing potential receiving >1 unit of D-positive WB or RBC	16/169 (9.5) [5.5-14.9]	25/285 (8.8) [5.8-12.7]	28/256 (10.9) [7.4-15.4]	64/382 (16.8) [13.1-20.9]	85/497 (17.1) [13.9-20.7]	.001

Abbreviations: RBC, red blood cell; WB, whole blood.

^a^ Unless otherwise indicated.

From its baseline (reference) proportion of 10.26% (95% CI 8.59%-12.13%) in 2020, WB use (eFigure 5 in Supplement 1) nearly tripled (RR 2.90, 95% CI 2.43-3.46, *P* < .001) to 29.78% (95% CI 28.22%-31.38%), whereas plasma-only transfusions remained relatively steady (ranging from a low of 11.01% (95% CI 9.84%-12.26%) in 2023 to a high of 14.29% (95% CI 12.68%-16.01%) in 2022. Initiation of multiple units decreased over time (*P* < .001), from a high of 51.56% (95% CI 48.67%-54.43%) in 2020 to a low of 44.43% (95% CI 42.72%-46.15%) in 2024. Completion of multiple units also decreased (*P* < .001) over time, from 41.80% (95% CI 38.98%-44.66%) in 2020 to 35.35% (95% CI 33.72%-37.02%) in 2024.

Compared with 2020, in 2024 females of childbearing potential were more likely (RR, 1.73; 95% CI, 1.45-2.80; *P* < .001) to receive D-positive WB or RBCs and also more likely to receive multiple D-positive units (RR, 1.81; 95% CI, 1.09-2.99; *P* = .02). Additional results for WB use and D-positive exposure in females of childbearing potential are provided in the eResults and eFigure 7 in Supplement 1.

## Discussion

CREDIT-EMS represents one of the first large-scale descriptions of nationwide US civilian PHT practices. We report nationwide 5-year PHT trends as well as interregional comparisons. Our results represent a useful addition to existing literature in a rapidly increasing arena of advanced prehospital care. Study findings illustrate the value of nationwide assessment of routinely practiced civilian PHT, which does not always mirror experiences from PHT trials or single-center evaluations.

A 2024 meta-analysis^6^ emphasized that plasma was the only blood product administered in 2 of the 3 civilian PHT RCTs. The mismatch between RCT study arms and routine PHT practices is clearly illustrated by our finding that plasma-only PHT represented only 12.1% of all cases.^15,16^ The movement away from plasma-only PHT is being accompanied by a marked increase in WB use across the country. From 2020 to 2024 the proportion of PHT cases receiving WB has tripled (from 10.0% to 30.0%). Compared with Midwest patients, those in the Northeast were nearly twice as likely to receive WB 33.2% (95% CI, 28.2%-38.6%, *P* < .001), vs 16.5% (95% CI, 14.6%-18.5%; *P* < .001).

The transition toward WB may, in part, be due to its suspected benefit over component therapy. A 2024 meta-analysis^28^ of available data included cautions regarding heterogeneity and bias but reported a WB survival benefit over component therapy. However, no such survival benefit was identified in another 2024 review and meta-analysis,^25^ published by the Eastern Association for the Surgery of Trauma (EAST); EAST’s conditional endorsement of WB was joined by only 7 of the panel’s 11 authors. Clinical justification for PHT’s move toward WB may be better evaluated after completion of TOWAR, TROOP, and/or PRIEST.

The increase in WB transfusions occurs in the setting of worldwide limitations in supply of D-negative type O blood.^21^ Our data demonstrate that as PHT shifts toward WB there is an accompanying increase in D-positive exposure to females of childbearing potential. The rate at which these females of childbearing potential are receiving D-positive WB or RBCs is on the rise, from 73 of 169 (43.20%, 95% CI 35.61%-51.02%) in 2020 to 372 of 497 (74.85%, 95% CI 70.79%-78.61%) in 2024. This rate of D-positive exposure is higher than previously seen in single-center reports (eg, 1 FCP every 30 months or 3-30 isoimmunized patients every 250 years^2^). Compared with 2020, in 2024 females of childbearing potential had a 73% increase in chances of receiving at least 1 (RR, 1.73; 95% CI, 1.45-2.80) or multiple (RR, 1.81; 95% CI, 1.09-2.99) units of D-positive blood product. Although proportions of females in the PHT population increased, females of childbearing potential accounted for a stable 15.2% of EMS transfusion cases. Others have estimated that 6% of patients receiving massive transfusion are FCP.^29^ Our data illustrate the need to avoid overextrapolation of massive transfusion or single-center data to the larger PHT experience.

The increasing exposure of females of childbearing potential to D-positive PHT should prompt further investigation of risk to benefit and patient preferences. Studies demonstrating patient acceptance of alloimmunization risks are informative but nondefinitive. Results from a survey^26^ providing a more precise estimate of PHT benefit (maternal mortality risk reduction of at least 4%) are helpful, but these findings should be considered preliminary due to low survey response rate (<7%) and identification of significant variation (eg, based on geography) in females of childbearing potential risk acceptance. Our results underline the need for further characterization of females of childbearing potential understanding and acceptance of alloimmunization risk. Patient-centered risk considerations and related health care resource issues should include alloimmunization topics beyond the actual occurrence of HDFN. Such issues can be classified as HDFN-related (eg, diagnosis, prevention, monitoring) and non-HDFN.

Our data provide no information on benefits associated with PHT. However, we find reason for optimism in 2 findings from the current data. First, in nearly all PHT cases the transport time exceeded 20 minutes, a previously proposed benchmark for PHT time-related benefit.^30,31^ Second, we found that PHT was not simply a pro forma matter of commencing transfusion a few minutes earlier. By an evidence-based^32^ surrogate end point of time savings, our findings suggest that PHT is effective in achieving timely delivery of a potentially important blood product.

All 3 RCTs evaluating civilian PHT address its application in trauma.^7,8,9^ Of the 3 ongoing PHT trials, the only one (PRIEST) that includes nontrauma does not include a nontransfused control group. Given our finding of frequent deployment of PHT for nontrauma, the common occurrence of nontrauma indications for PHT,^33^ and others^2^ who are expanding civilian PHT to include nontrauma cases (eg, obstetric hemorrhage), future studies should assess outcomes associated with PHT in nontrauma.

### Limitations

This study has limitations. The dataset was large but had a narrow focus, which limited conclusions. CREDIT-EMS results provide only introductory insights; further research could include advanced modeling controlling for factors such as region × time interactions. Time-based variables could also be more meaningfully assessed with more years of data.

For conclusions regarding geographic disparity, selection of US Census Bureau regions represented an attempt to broadly compare PHT in those cases who received PHT (not just those who may have been eligible). A more granular geographic partitioning could yield different results. Even within the same area, different medical direction preferences and protocols could account for differences in EMS practices.

In painting a large-scale picture of PHT in the US, CREDIT-EMS did not delve into patient-level details. The study did not have sufficient information on patient diagnosis or situational findings to allow conclusions to be drawn around PHT indications. The subject of PHT for nontrauma, for instance, was not able to be evaluated in detail and remains a potential focus for future work.

An additional shortcoming of our analysis is that we had limited follow-up information. This issue translated into an inability to draw conclusions regarding clinical effect of findings, such as D-positive blood exposure, in females of childbearing potential. Longitudinal research is needed to quantify the community effects of such exposure.

## Conclusions

The current study represents a broad description of civilian PHT in the US. The findings are a potentially useful contribution to the state of understanding of both transfusions and recipients of PHT. These findings also highlight the need for standardized protocols and further evaluation of risk-benefit considerations.

## Notes

Supplement 1. **eFigure 1.** Geographic regions of the United States classified by the US Census Bureau. Each region (Northeast, Midwest, South, and West) is labeled and color-coded to distinguish the number of states within each region. **eFigure 2.** Representation of US states included in the CREDIT-EMS study. States’ enrollment *n* is listed and categorized into quartiles with darker shading corresponding to higher quartile. **eFigure 3.** Composition of the 17 927 blood product units transfused during prehospital care. The figure uses color-coded segments to depict the relative proportions of whole blood, red blood cells, plasma, and other blood products. **eFigure 4.** Temporal trends in the proportion of female transfusion recipients, stratified into three categories: all ages, ages 12–50 (females of childbearing potential), and ages 0–50. The figure uses distinct line markers to highlight changes over the study period. **eFigure 5.** Proportions of prehospital transfusions that included whole blood from 2020 to 2024. Bars are color-coded by year to demonstrate the increasing utilization of whole blood, rising from 10% to 30%. **eFigure 6.** Proportions of prehospital transfusion cases involving multiple units. Data are shown for both units initiated and completed before hospital arrival, with separate bars representing each category and year. **eFigure 7.** Trends in the proportion of females of childbearing potential (ages 12–50) receiving D-positive blood products during prehospital transfusions. Lines are differentiated to show the rising proportions over the study period.

Supplement 2.
Data Sharing Statement
