# Supplementary material for: Characteristics, Regional Evaluation, and D-Antigen in Transfusions by Emergency Medical Services
Source: JAMA Netw Open. 2025 Jul 31;8(7):e2524368. doi: 10.1001/jamanetworkopen.2025.24368 (PMC12314721; doi:10.1001/jamanetworkopen.2025.24368)
Supplement: Supplement 1. — eFigure 1. Geographic regions of the United States classified by the US Census Bureau. Each region (Northeast, Midwest, South, and West) is labeled and color-coded to distinguish the number of states within each region. eFigure 2. Representation of US states included in the CREDIT-EMS study. States’ enrollment n is listed and categorized into quartiles with darker shading corresponding to higher quartile. eFigure 3. Composition of the 17 927 blood product units transfused during prehospital care. The figure uses color-coded segments to depict the relative proportions of whole blood, red blood cells, plasma, and other blood products. eFigure 4. Temporal trends in the proportion of female transfusion recipients, stratified into three categories: all ages, ages 12–50 (females of childbearing potential), and ages 0–50. The figure uses distinct line markers to highlight changes over the study period. eFigure 5. Proportions of prehospital transfusions that included whole blood from 2020 to 2024. Bars are color-coded by year to demonstrate the increasing utilization of whole blood, rising from 10% to 30%. eFigure 6. Proportions of prehospital transfusion cases involving multiple units. Data are shown for both units initiated and completed before hospital arrival, with separate bars representing each category and year. eFigure 7. Trends in the proportion of females of childbearing potential (ages 12–50) receiving D-positive blood products during prehospital transfusions. Lines are differentiated to show the rising proportions over the study period. [file jamanetwopen-e2524368-s001.pdf]

Supplemental Online Content

Rosen CL, Thomas SA, McCartin MP, et al. Characteristics, regional evaluation, and D-antigen in transfusions by emergency medical services: the CREDIT-EMS study. *JAMA Netw Open*. 2025;8(7):e2524368. doi:10.1001/jamanetworkopen.2025.24368

eAppendix 1. Study Setting Details

eAppendix 2. Extended Results

eFigure 1. US Census Bureau Regions

eFigure 2. States Represented in CREDIT EMS

eFigure 3. Composition of Blood Products

eFigure 4. Female Proportions of PHT Cases by Year

eFigure 5. Yearly Proportion of PHTs That Include WB

eFigure 6. Yearly Proportion of PHTs That Include Initiation of Multiple Units of Blood

eFigure 7. Yearly Proportion of FCPs Receiving D-Positive Blood Products

eTable 1. Geographic Regions and Transport Times

eTable 2. Geographic Regions and Median Ages of Females of Childbearing Potential

This supplemental material has been provided by the authors to give readers additional information about their work.

eAppendix 1. Study setting details

This section provides extended information on CREDIT EMS participating services. While study participants from the US employed varying PHT protocols that often differed even within the same state, the usual approach for WB or RBCs was either low-titer Group O+ WB or leukoreduced RBC. None of the PHT provided in CREDIT EMS included plasma derivatives other than liquid plasma, FFP, or cryoprecipitate.

Extended information on US study sites: geography

Patients were geographically classified based on their destination hospital's US Census Bureau region. As depicted in eFigure 1, there are four regions: Northeast, Midwest, South (16 states plus the District of Columbia), and West. eFigure 2 depicts the states represented in CREDIT EMS.

Extended information on US study sites: EMS programs

Most of the study's data came from two data sources representing commercial EMS providers: Air Methods and Global Medical Response. These providers' PHT services are largely focused on helicopter EMS but also include fixed-wing and ground transport. Between the two commercial providers there are approximately 500 EMS bases covering all 50 states. PHT protocols vary between and within the commercial providers' jurisdictions.

One commercial provider (Air Methods) transports over 100,000 patients annually across 48 states. Clinical care is provided by nurses and paramedics staffing mostly helicopter EMS units at 150 bases. PHT in different geographic areas may include WB, packed RBCs, liquid plasma, fresh frozen plasma, cryoprecipitate, or platelets.

The second commercial provider (Global Medical Response) operates many smaller organizations providing air and ground transport from 375 bases in 38 states. The nurse and paramedic crews' PHT options in different regions may include WB, packed RBCs, liquid plasma, fresh frozen plasma, or platelets.

The third provider of CREDIT EMS data was EMSA, the public-service EMS authority for the 1.7 million inhabitants of Tulsa and Oklahoma City. EMSA's paramedic crews use a PHT protocol consisting of low-titer O+ WB transfusion. From the protocol's institution at the end of 2022 (data for this study were contributed for 2023 and 2024) through the latter half of 2024, PHT was restricted to males and females at least 51 years of age; PHT was approved for use in females of potential childbearing age in July 2024.

The final three CREDIT EMS data sources are non-profit programs with similar staffing (usually a nurse and paramedic) and similar PHT approaches. Data were contributed by one university-affiliated service (Vanderbilt LifeFlight), one regional service (Boston MedFlight), and one statewide non-profit (LifeFlight of Maine).

Vanderbilt LifeFlight operates eight rotor-wing bases, one fixed-wing base, and one critical care ground base. Each year the program transports approximately 4,400 patients (3/4^{ths} by air). The PHT protocol includes liquid (not thawed) plasma and packed RBCs.

Boston MedFlight transports 6,100 patients annually using five helicopters, three ground vehicles, and a fixed-wing aircraft. PHT cases, who received packed RBCs and/or plasma, were accrued into this study if they were transported to the study's base hospital (Beth Israel Deaconess Medical Center).

LifeFlight of Maine operates at three bases and transports over 2,500 patients annually using five helicopters, three ground vehicles, and a fixed-wing aircraft. PHT capabilities include WB, packed RBCs, and liquid plasma.

© 2025 Rosen CL et al. *JAMA Network Open*.

eAppendix 2. Extended Results

Transport times

Prehospital times were not recorded in 101 patients; the denominator for time calculations was thus 10,343. In at least one case with no prehospital time, a CREDIT EMS study agency initiated PHT but the patient was transported by a different (non-study) EMS agency. For another 16 patients lacking documented transport time there was specific notation of scene death. There was no explicit documentation of explanation for missingness in the other 84 cases, but these likely involved scene death. (*N.B.* None of the missing-time cases that likely died were from the three data sources that provided CREDIT EMS' mortality data.)

Kruskal-Wallis testing identified a significant ($p < .001$) association between US region and transport time. *Post hoc* testing identified each inter-regional comparison as significant (all pairwise $p < .001$). eTable 1 depicts effect size estimation for the median inter-regional differences. Since we chose a conservative approach of clustering the Hodges-Lehmann estimator on data source (*i.e.* up to six clusters) some 95% CIs overlapped the null despite significant pairwise p values.

eTable 1. Geographic regions and transport times

Region	Median (IQR) transport minutes	Comparator	Median difference (95% CI)
Northeast	56 (40-80)	Midwest	6 (-14 to 30)
		South	9 (-6 to 27)
		West	13 (-6 to 36)
Midwest	48 (38-65)	South	9 (5 to 13) [a]
		West	16 (16 to 16) [a]
South	46 (35-61)	West	20 (18 to 22) [a]
West	65 (44-103)	-	-

Abbreviations: CI – confidence interval; IQR – interquartile range

[a] Non-overlap of null value (consistent with significant difference at $p < .05$ level)

We also report as preliminary (due to low n) the prehospital times for patients transported by ground as compared to air. The air group had a median transport time of 48 minutes (IQR 36-64), which was significantly ($p < .001$) longer than the corresponding time for PHT cases transported by ground (median 33, IQR 23-56). For the ground EMS–only data source, the median transport time was 25 minutes with IQR 20-29. PHT cases from this data source had at least one unit of blood product completed prior to hospital arrival in 84.0% of cases (similar to the overall study rate of 80.1%).

Regional ages of FCPs receiving PHT

eTable 2 presents summary statistics for ages of the FCP patients in the four US regions. By definition, the ranges in all regions were bounded by ages 12 and 50. Since the overall Kruskal-Wallis assessment suggested a significant (p = .006) difference between ages, pairwise comparisons are reported in the table. Within the US, Northeast FCPs were significantly older than those in the Midwest (p = .010) or South (p = .004).

eTable 2. Geographic regions and median ages of females of childbearing potential

Region	Median (IQR) age of FCP	Comparator	Median difference (95% CI)

© 2025 Rosen CL et al. *JAMA Network Open*.

Northeast	39 (30-46)	Midwest	5 (2 to 9) [a]
		South	6 (2 to 9) [a]
		West	4 (1 to 8)
Midwest	32 (22-40)	South	0 (-2 to 2)
		West	1 (-1 to 3)
South	32 (23-41)	West	1 (-1 to 3)
West	34 (24-40)	-	-

Abbreviations: FCP – females of childbearing potential (age 12-50); CI – confidence interval; IQR – interquartile range

[a] Non-overlap of null value (consistent with significant difference at $p < .05$ level)

Transfusions & components

eFigure 3 demonstrates a blood product-based unit of analysis for PHT cases in this study. The types of transfusions (n = 17,927) are depicted, with additional breakdown of the three blood products (fresh frozen plasma, liquid non-frozen plasma, and cryoprecipitate) comprising CREDIT EMS' "plasma" category.

Pairwise comparisons for initiation and completion of multiple units of transfusion

As outlined in Table 3 of the main results, the South's rates of both initiation and completion of multiple units of transfusion were higher than corresponding rates in any of the other regions (all pairwise $p < .001$). For assessment of regional proportions of PHTs with multiple units initiated, the only other statistically significant finding was a higher proportion in the Midwest as compared to the West.

For completion of multiple units of blood product prior to hospital arrival, the South and Midwest regions had similar rates (p = .214), while the rates in the West and Northeast were similar (p = .438). The South and Midwest rates were higher than those seen in either the West ($p < .001$ for both comparisons) or the Northeast (p = .005 for South *vs.* Northeast, p = .043 for Midwest *vs.* Northeast).

Proportions of females and FCP by analysis year

The upward trend in numbers of (all-age) females was not present in either FCP or in females aged 0-50. eFigure 4 depicts the annual proportions of three groupings of females: all-ages, 12-50, and 0-50. Adding females aged 0-11 to the tabulation of FCPs has a small effect: the overall n of FCP changed from 1,598 to 1,664 and each of the US regions' change in absolute percentage of FCP increased by 0.6% (Northeast and South) or 0.7% (Midwest and West). The lack of temporal trend in FCP over the study period was also noted when females aged 0-11 were added to the tabulation of FCP (p for trend = .316).

Other results by analysis year

As detailed in the main Results, additional analyses by year included proportion of PHT including WB, proportion of PHT entailing institution of multiple units of transfusion, and proportion of FCPs receiving D-positive blood products. These results are depicted in eFigures 5-7.

© 2025 Rosen CL et al. *JAMA Network Open*.

eFigure 1. Geographic regions of the United States classified by the U.S. Census Bureau. Each region (Northeast, Midwest, South, and West) is labeled and color-coded to distinguish the number of states within each region.

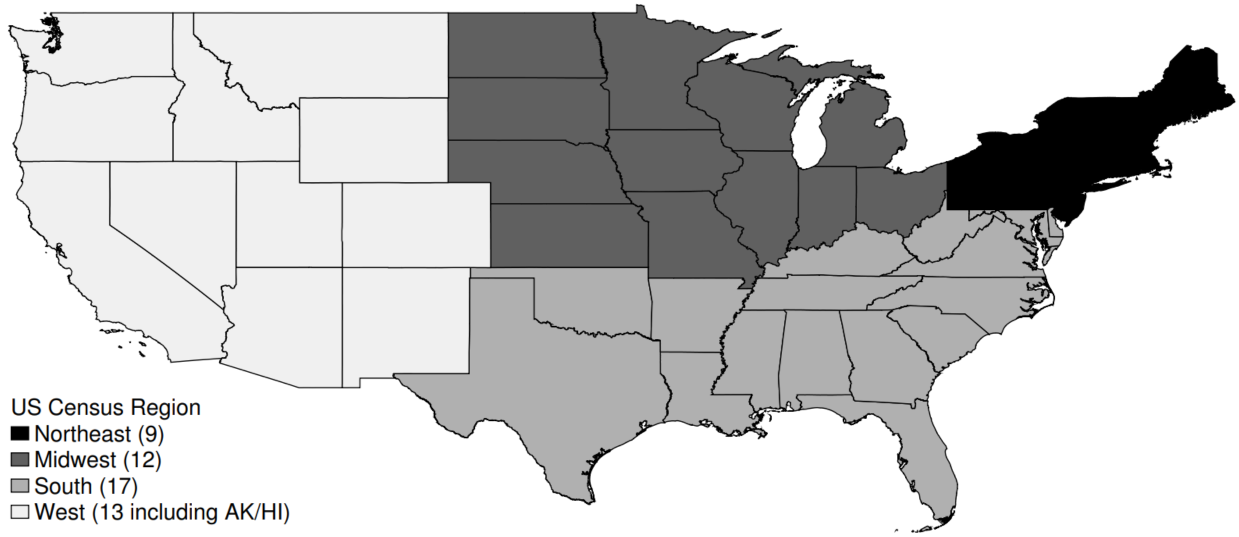

© 2025 Rosen CL et al. *JAMA Network Open*.

eFigure 2. Representation of U.S. states included in the CREDIT-EMS study. States' enrollment *n* is listed and categorized into quartiles with darker shading corresponding to higher quartile.

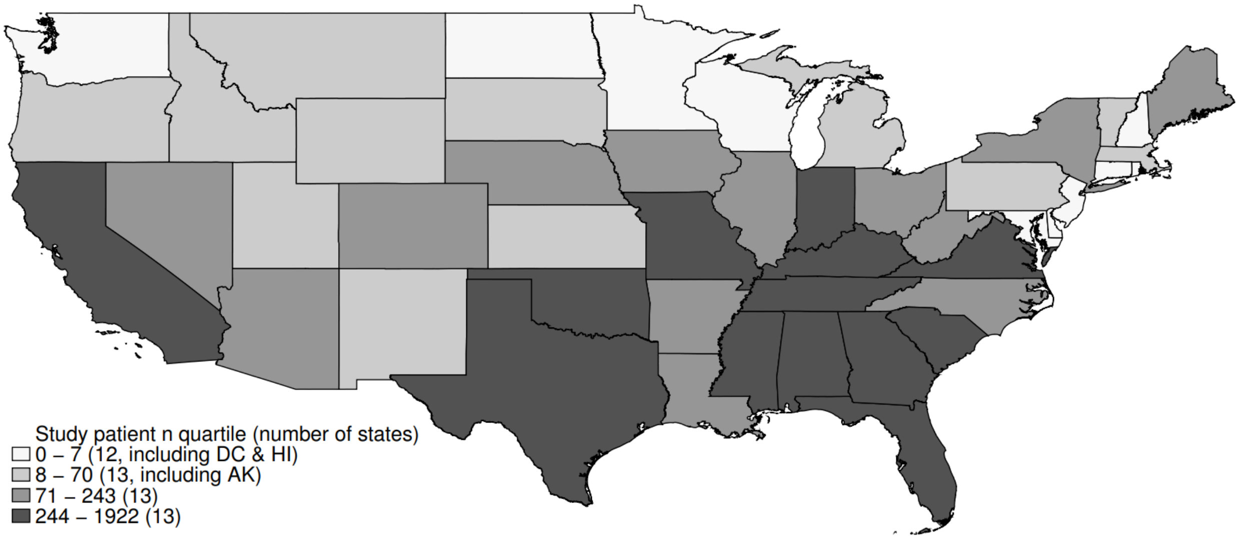

© 2025 Rosen CL et al. *JAMA Network Open*.

eFigure 3. Composition of the 17,927 blood product units transfused during prehospital care. The figure uses color-coded segments to depict the relative proportions of whole blood, red blood cells, plasma, and other blood products.

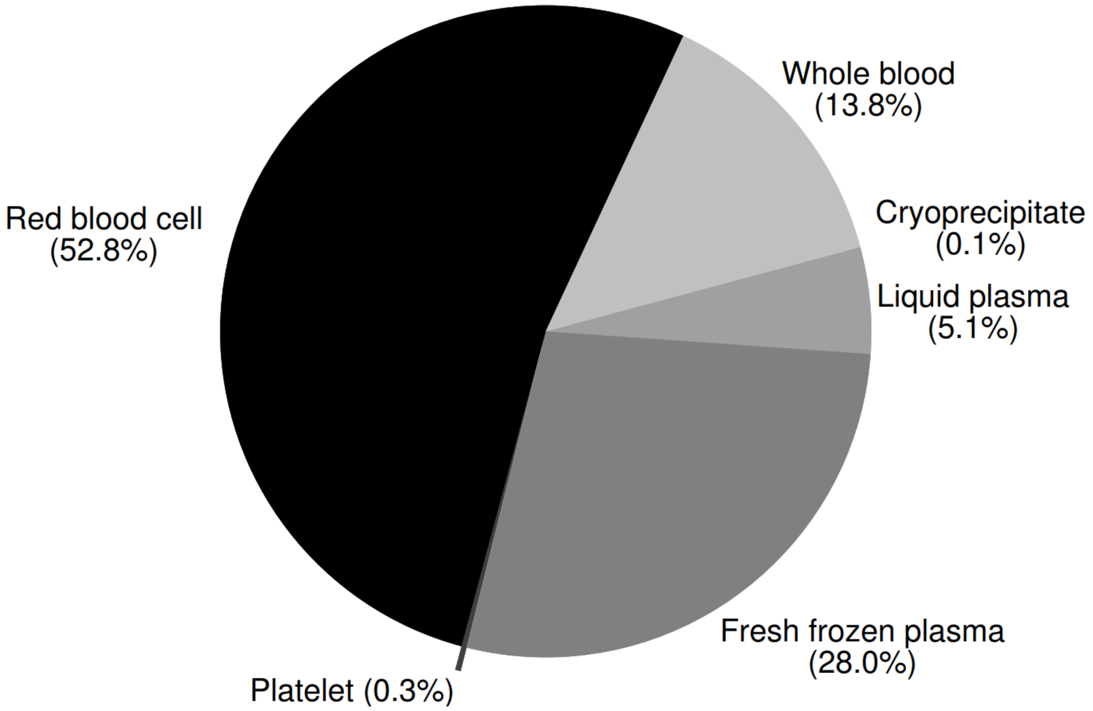

© 2025 Rosen CL et al. *JAMA Network Open*.

eFigure 4. Temporal trends in the proportion of female transfusion recipients, stratified into three categories: all ages, ages 12–50 (females of childbearing potential), and ages 0–50. The figure uses distinct line markers to highlight changes over the study period.

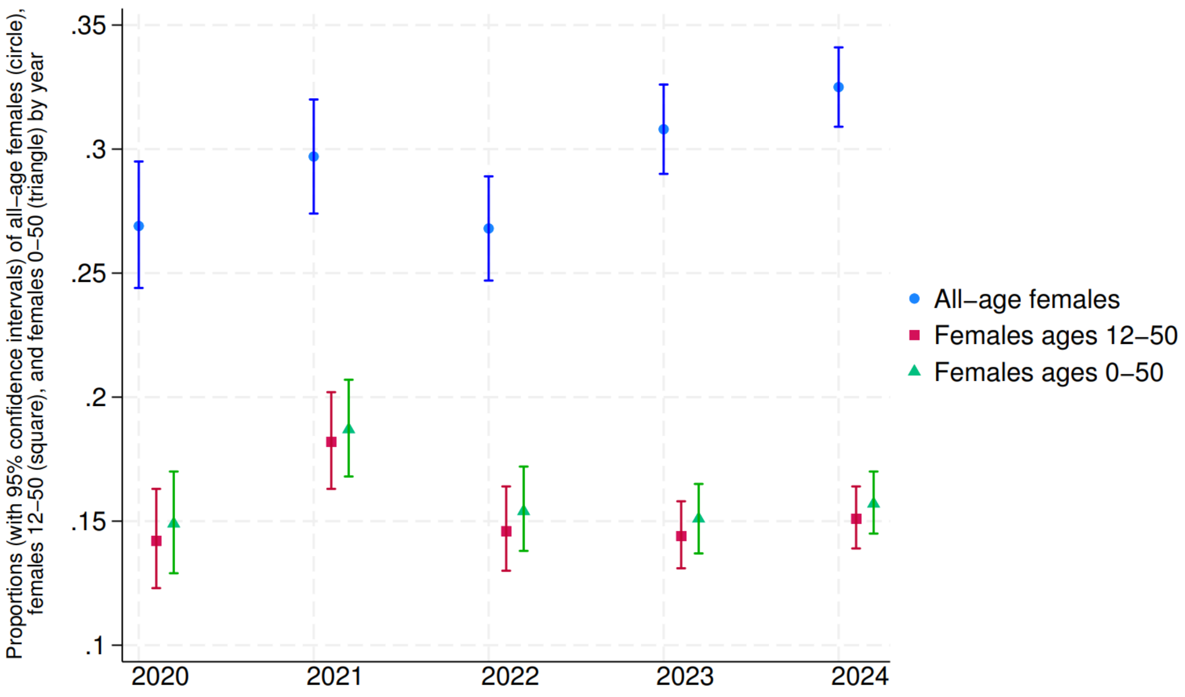

© 2025 Rosen CL et al. *JAMA Network Open*.

eFigure 5. Proportions of prehospital transfusions that included whole blood from 2020 to 2024. Bars are color-coded by year to demonstrate the increasing utilization of whole blood, rising from 10% to 30%.

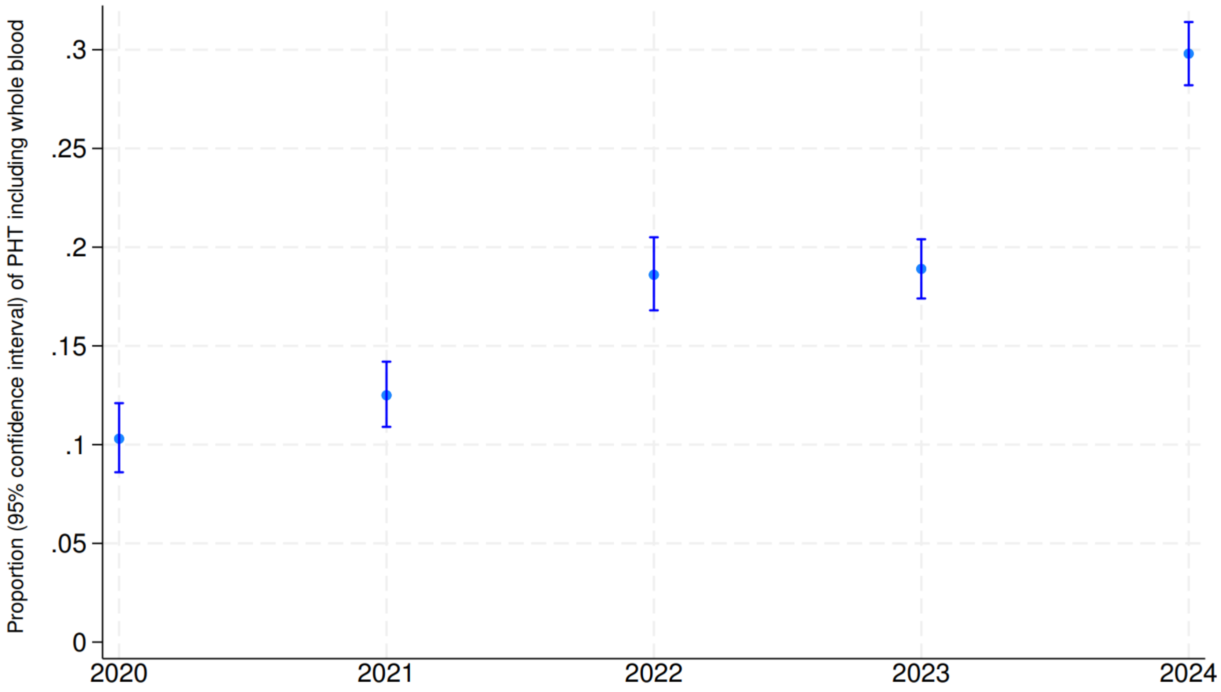

© 2025 Rosen CL et al. *JAMA Network Open*.

eFigure 6. Proportions of prehospital transfusion cases involving multiple units. Data are shown for both units initiated and completed before hospital arrival, with separate bars representing each category and year.

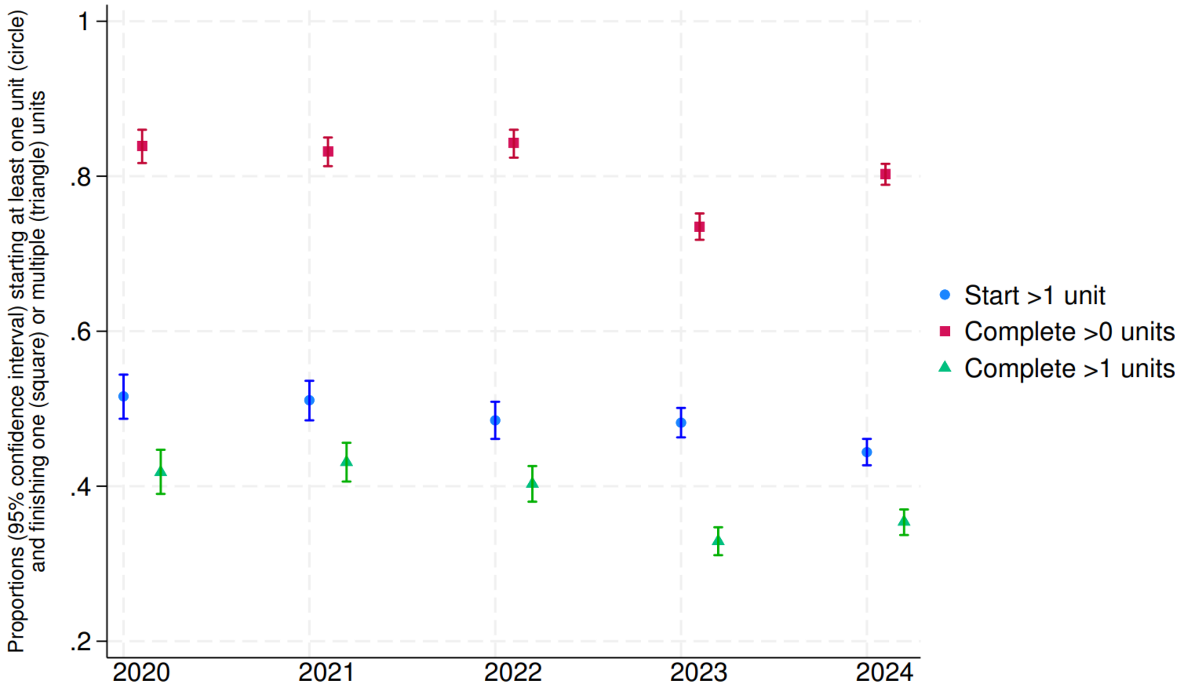

© 2025 Rosen CL et al. *JAMA Network Open*.

eFigure 7. Trends in the proportion of females of childbearing potential (ages 12–50) receiving D-positive blood products during prehospital transfusions. Lines are differentiated to show the rising proportions over the study period.

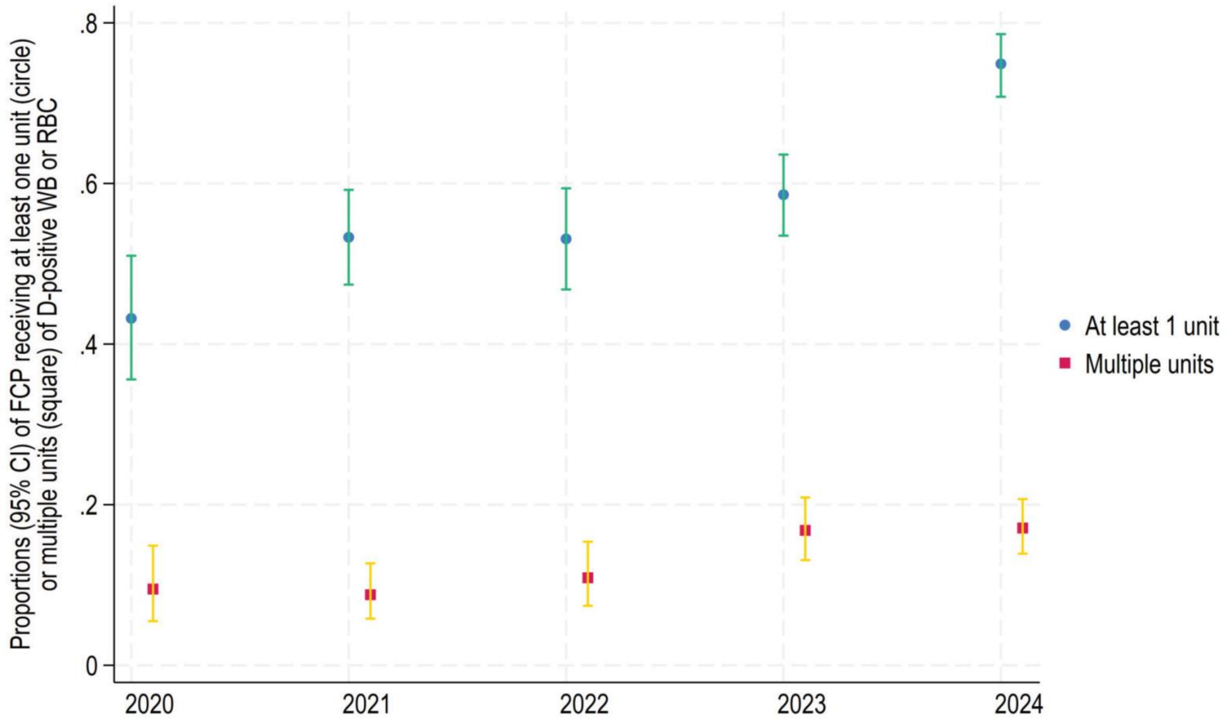

© 2025 Rosen CL et al. *JAMA Network Open*.
