# Supplementary material for: Characteristics, Regional Evaluation, and D-Antigen in Transfusions by Emergency Medical Services
Source: JAMA Netw Open. 2025 Jul 31;8(7):e2524368. doi: 10.1001/jamanetworkopen.2025.24368 (PMC12314721; doi:10.1001/jamanetworkopen.2025.24368)
Supplement: Supplement 2. — Data Sharing Statement [file jamanetwopen-e2524368-s002.pdf]

Data Sharing Statement

Rosen. Characteristics, Regional Evaluation, and D-Antigen in Transfusions by Emergency Medical Services. *JAMA Netw Open*. Published July 31, 2025.
doi:10.1001/jamanetworkopen.2025.24368

Data
Data available: No
